# Crossover of quasi-localized dynamics and diffusion in supercooled liquids

**DOI:** 10.1038/s41567-026-03320-5

**Published:** 2026-06-09

**Authors:** Federico Caporaletti, Simone Capaccioli, Dimitrios Bessas, Aleksandr I. Chumakov, Alessandro Martinelli, Francesco Dallari, Giulio Monaco

**Affiliations:** 1 Laboratory of Polymer and Soft Matter Dynamics, Experimental Soft Matter and Thermal Physics, Université libre de Bruxelles, Brussels, Belgium; 2 Physics Department, University of Pisa, Pisa, Italy; 3 European Synchrotron Radiation Facility, Grenoble, France; 4 Department of Physics and Astronomy ‘Galileo Galilei’, University of Padova, Padova, Italy; 5 Present Address: Sustainable Materials Engineering (SUME), Vrije Universiteit Brussel, Brussels, Belgium; 6 Present Address: Laboratoire Charles Coulomb, Université de Montpellier, CNRS, Montpellier, France

**Keywords:** Structure of solids and liquids, Statistical physics, Glasses

## Abstract

Secondary relaxation, also known as β_JG_ relaxation, is a dynamical process observed in supercooled liquids and glasses. It is distinct from the slower, so-called α relaxation, which is associated with particles escaping the cages formed by their surrounding neighbours. The secondary relaxation affects several properties of the glass, including the crystallization propensity and mechanical response. Traditionally studied via dielectric and mechanical spectroscopy, the β_JG_ relaxation is often linked to quasi-localized particle motion. Here we use a wavevector-resolved X-ray technique to access microscopic, real-space information on the β_JG_ relaxation in a hydrogen-bonded glass former and show that the α and β_JG_ relaxations cannot be described as two independent processes. Moreover, we provide evidence that the β_JG_ relaxation is associated with a sublinear growth of the mean-squared molecular displacement preceding the onset of diffusion. Our findings thus clarify that the β_JG_ relaxation can be described as the critical rattling of molecules within the cage formed by their neighbours just before escaping from it.

## Abstract (web summary)

Relaxation processes in glasses display two distinct components—fast localized motions versus slower diffusive dynamics. Time-domain interferometry experiments with a typical glass former now show that the two processes are coupled.

## Main

A liquid cooled sufficiently fast below the melting temperature does not crystallize and can be deeply undercooled until it eventually reaches the glass state across the glass-transition temperature *T*_g_ (ref. ^1^). Upon cooling, the molecular dynamics become slower and slower, and around a temperature often identified with the mode-coupling transition temperature *T*_c_ ≈ 1.2*T*_g_, a plateau develops in the time dependence of the mean-squared molecular displacement^2,3^. This plateau is a signature of caged dynamics: the molecules remain trapped in the cage formed by their first neighbours (the plateau in the mean-squared displacement) before escaping and diffusing away. *T*_c_ also marks the temperature below which a secondary relaxation process, known as Johari–Goldstein or β_JG_ relaxation^4^, branches off the slower, main relaxation process, known as α or structural relaxation^1^. The β_JG_ relaxation is still under investigation and, despite appearing in the temperature range below *T*_c_ crucial for a detailed understanding of the glass-transition, its role in the vitrification process remains under debate^5–7^.

Johari and Goldstein were the first to report evidence of β_JG_ relaxation in the dielectric spectra of glass formers composed of rigid molecules^4^. A large literature of dielectric spectroscopy studies of mainly organic glass formers has been accumulated since then^8,9^, and criteria have been proposed to distinguish ‘genuine’ β_JG_ relaxations from processes related to internal degrees of freedom^10^. Although the β_JG_ relaxation was initially considered purely rotational in nature due to its appearance in dielectric spectroscopy spectra^11^, its observation by techniques probing density fluctuations^12^ and in mechanical spectroscopy studies of metallic glasses^13^, where no orientation degrees of freedom are present, suggests a substantial translational component. An important outcome of the available studies has been a comprehensive understanding of the role of the β_JG_ relaxation in a number of relevant mechanical properties such as, for example, the plastic response of the material^13^. More recently, calorimetry has also been used to characterize the β_JG_ process, adding an additional perspective^14,15^.

Different conceptual frameworks, not clearly compatible with each other, have been used to describe the β_JG_ relaxation. In a real-space picture, it is usually interpreted as a quasi-localized process occurring in loosely connected regions of the glass structure^4^. This description is supported by confocal microscopy experiments, where clusters of fast-moving particles could be directly observed in colloidal supercooled fluids and glasses^16^. Although real-space imaging of bulk liquids with atomic resolution is still very difficult, consistent results were obtained in experiments probing the structure of metallic glasses^17^. One- and two-dimensional ^2^H-nuclear magnetic resonance (NMR) studies provided evidence that the secondary relaxation is associated with small-angle reorientational jumps, which involve essentially all molecules over time^18,19^. In the coupling model, the β_JG_ relaxation is associated to the dynamics of particles that escape from their cage and is considered to be the precursor of the α relaxation^20^. In the potential energy landscape framework^21,22^, the β_JG_ relaxation is associated with transitions between neighbouring minima with small energy barriers, which corrugate larger ‘metabasins’ explored during the α relaxation. This topographic hierarchy in the potential energy landscape has been supported by a recent numerical simulation of an asymmetric dimer system^23^. However, a hierarchical separation between basins of different heights does not seem to be necessarily required. Within random first-order transition theory, for example, the β_JG_ relaxation is associated to a low free-energy tail of the activation-barrier distribution, which emerges because of the differences in the geometry of the reconfiguring regions related to the α and β_JG_ relaxations^24^. Numerical simulations covering exceptionally long times instead support a scenario in which the β_JG_ relaxation originates from a heterogeneous activated dynamics with dynamic facilitation, a phenomenon in which microscopic motion induces further motion nearby^25,26^.

Despite a large amount of work that has already been devoted to this subject, pinpointing the microscopic characteristics of the β_JG_ relaxation process, for example, the rotational versus translational molecular displacements involved in it and their distribution in space and time, remains an extraordinary theoretical, numerical and experimental challenge. We here contribute to this matter by reporting an experimental study of the wavenumber-resolved dynamical response of the β_JG_ relaxation in a model hydrogen-bonded liquid, 5-methyl-2-hexanol (5M2H), in the temperature range where it separates from the α relaxation. The data collected, analysed using a new inversion approach, put us in a position to provide a clear picture of the β_JG_ relaxation: it corresponds to the critical rattling of the molecules in the cage formed by their first neighbours, triggering the onset of diffusion. This work thus identifies the microscopic motions that signal the onset of glassy behaviour.

**Fig. 1:**
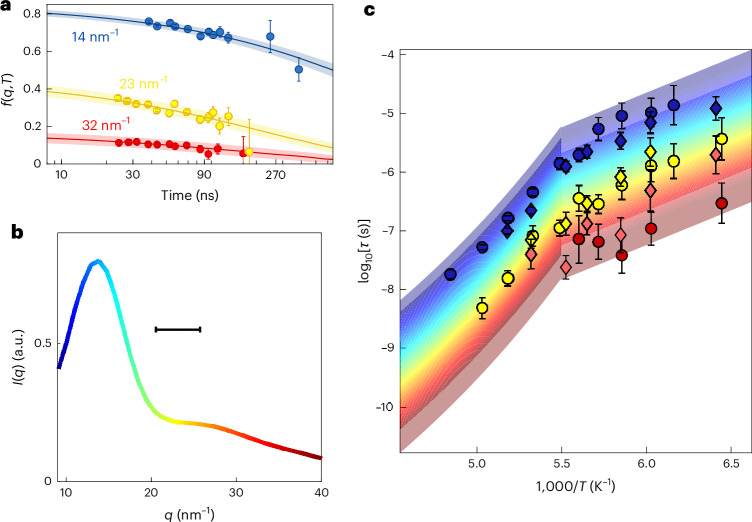
Nuclear γ-resonance time-domain experimental results. **a**, Intermediate scattering functions extracted from γ-resonance TDI measured at 176.9 K at the indicated wavenumbers. The solid lines show best fits to the data using the KWW function (equation (1)). The error bars denote ±1 s.d., computed after fitting and reduction of the TDI beating patterns (ref. ^56^ and Supplementary Text 1) and subsequent logarithmic binning on the time axis. Each bin contains *n* = 11 time delays for *t* < 60 ns, increasing to *n* = 22 for 60 < *t* < 120 ns and to *n* > 100 at longer times owing to reduced count rates. The s.d. combines the within-bin variance with propagated uncertainties from the data reduction procedure. Each beating pattern has a total integrated intensity >1 × 10^5^ counts, with an average of >50 counts per time delay (Supplementary Fig. 1). Shaded regions indicate the 68% confidence intervals obtained from the fitting procedure. **b**, Total scattered intensity measured at 176.9 K. The covered *q*-interval ranges from 9 to 40 nm^−1^ and is associated to the shown range of colours. The horizontal bar indicates the typical wavenumber resolution in the experiment. **c**, The diamonds represent the temperature dependence of the relaxation time, *τ*, measured at different wavenumbers identified according to the colours in **b**. In the plot, we also report data obtained in a previous experiment (circles)^29^. Error bars are ±1 s.d., obtained from the weighted nonlinear fitting of the experimental data^57^.

In the nuclear γ-resonance time-domain experiment that we discuss here (see Supplementary Fig. 1 for an example of raw data), it is possible to extract the (normalized) intermediate scattering function, *f*(**q**, *t*), that is the *q*-component of the (normalized) density correlation function at time *t* (ref. ^27^) (see Methods and Supplementary Text 1 for more details). This technique is used here to study the microscopic density fluctuations in deeply supercooled 5M2H, a model hydrogen-bonded glass former characterized by a genuine β_JG_ process^28^ and with a characteristic timescale ideal for the dynamic range of time-domain interferograms (TDI)^29^. Figure 1a reports the intermediate scattering function of 5M2H measured at 176.9 K as a function of time for three *q*-values (see Fig. 1b for the corresponding total scattering profile). The decay with time of density fluctuations is well described by a stretched exponential, also called the Kohlrausch-Williams-Watts (KWW) function, as in previous studies^12,29,30^

1 $$f({\bf{q}},t)={f}_{{\bf{q}}}(T)\exp \left[-{\left(\frac{t}{\tau ({\bf{q}},T)}\right)}^{{\beta }_{\mathrm{KWW}}}\right].$$

By fitting equation (1) to the experimental data of *f*(**q**, *t*) for different temperatures and wavenumbers, as shown in the examples reported in Fig. 1a, the relaxation time, *τ*(*q*, *T*), and the relaxation strength, *f*_*q*_(*T*), have been extracted. The shape parameter, *β*_KWW_, has been fixed to 0.5 for all temperatures and *q* to reduce the number of free fitting parameters. However, the results of this study are not affected by variations of *β*_KWW_ in the ±0.2 range (see Supplementary Text 2, Supplementary Fig. 2 and Supplementary Table 1 for justification and more details).

**Fig. 2:**
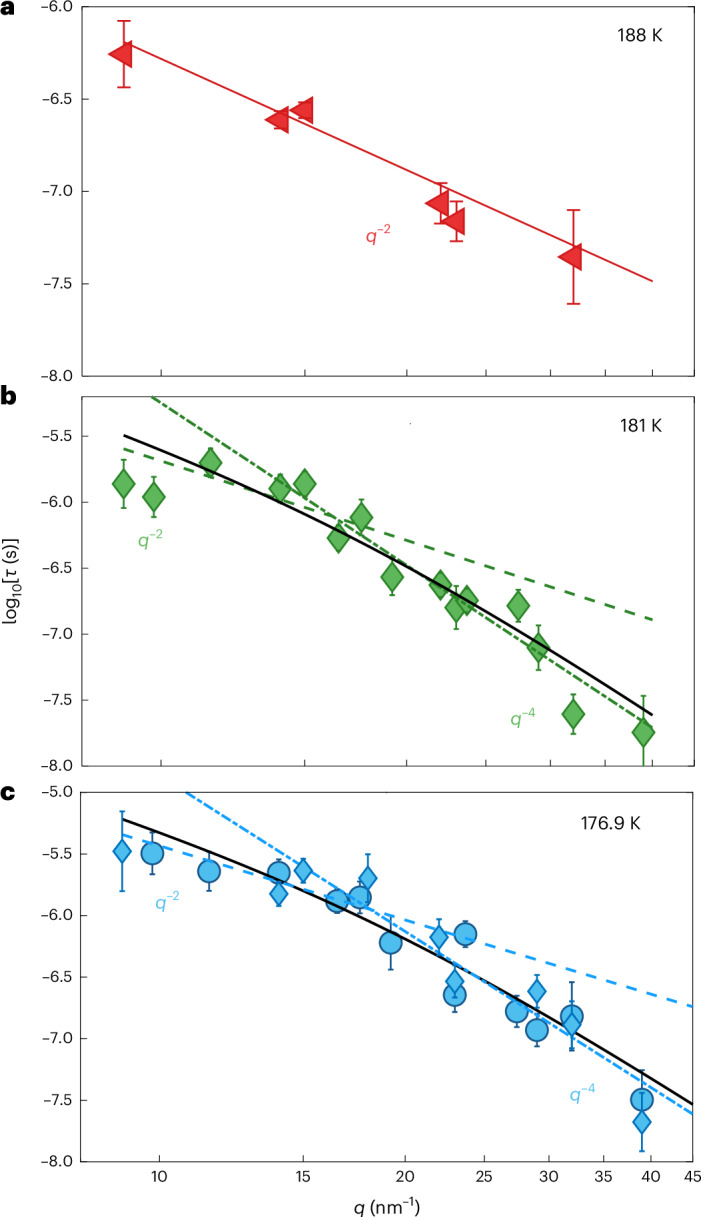
The α-to-β_JG_ transition observed as a function of *q*. **a**–**c**, Wavenumber dependence of the relaxation time at 188 K (**a**), 181 K (**b**) and 176.9 K (**c**) across *T*_αβ_ ≈ 181 K (ref. ^29^), as reported in the legends. The dashed and dashed-dotted lines through the data for *τ* at *T* = 181 K (**b**) and *T* = 176.9 K (**c**) show, at *q* ≈ 16 nm^−1^, a transition from a *q*^−2^ regime at low *q* to a *q*^−4^ regime at high *q*. The full lines through the data correspond to equation (2). The data for *τ* at *T* = 188 K (**a**) only show the *q*^−2^ regime. The error bars denote ±1 s.d. from the nonlinear fitting procedure of the beating patterns.

The temperature dependence of *τ*, probed at different *q*, is shown in the relaxation map reported in Fig. 1c. As already observed in ref. ^29^, a change in the activation energy of the relaxation time can be observed around *T*_αβ_ = 181 K, with the activation energy matching that of the α relaxation above *T*_αβ_ and that of the β_JG_ relaxation below *T*_αβ_ (ref. ^12^). In the present work, we extend our previous investigation reported in ref. ^29^ to a larger *q*-range and to a denser *q*-grid, as shown in Fig. 2, for the *q-*dependence of the relaxation time.

Above *T*_αβ_, we observe in Fig. 2a an inversely quadratic wavenumber dependence for *τ*, compatible with the diffusive behaviour expected in this region, as also reported for other glass formers^31^. Across *T*_αβ_, a clear change in the *q*-dependence of *τ* can be observed (Fig. 2b,c). In more detail, at both 181 K and 179.6 K and at scattering vectors larger than ~16 nm^−1^, *τ* displays, in agreement with previous observations, a super-quadratic *q*-dependence, *τ* ∝ *q*^−*n*^ with *n* > 2, which is a hallmark of restricted subdiffusive dynamics. In particular, the value of the exponent *n* averaged over the data at 181 K and 176.9 K is 〈*n*〉 = 4.3(3). Instead, at smaller *q*-values, an inversely quadratic *q*-dependence is observed, indicating that the diffusive dynamics is recovered at small scattering vectors (that is, large distances and long times). As already reported in our previous study^29^ and here even more clearly demonstrated, the wavenumber at which the *q*-dependence changes from diffusive to subdiffusive, *q*_DS_ ≈ 16 nm^−1^, corresponds to a root mean-squared displacement $\sqrt{6}/{q}_{\mathrm{DS}}\simeq 1.5\,\mathring{\rm A}$, which is ~20% of the average intermolecular distance. This value is compatible with Lindemann’s criterion for structural instability and with the observation that, in simulated metallic glasses, the α relaxation appears when molecular displacements reach roughly 20% of the distance of nearest neighbours^32^. From a different perspective, we also observe that the visibility of the β_JG_ relaxation in the density correlation function at high *q* might be explained by thinking that this process has a strong rotational component that dominates the density correlation function at high *q*, as the rotational–translational coupling becomes strong there, consistent with the results of a molecular dynamics study of undercooled water^33^. The transition between the two different regimes for *τ* is smooth and can be described using the simple expression

2 $$\tau (q)=\frac{1}{{D}_{{\rm{\alpha }}}{q}^{2}+{\widetilde{D}}^{2}{q}^{4}},$$

where *D*_α_ is the diffusion coefficient, and $\widetilde{D}$ is an anomalous diffusion coefficient associated with the subdiffusive dynamics within the β_JG_ relaxation^29,34^. The full lines through the experimental data at 181 K and 176.9 K in Fig. 2 are the results of a fit using equation (2) and describe the data rather well. At first glance, this result could be interpreted, following a study on dielectric spectroscopy and neutron scattering experiments^34,35^, assuming that density fluctuations around the decoupling temperature *T*_αβ_ relax via two independent relaxation channels, the α and the β_JG_ ones, which exist and are active at all *q*. The intermediate scattering function could then be written as the product of the two relaxation processes: *f*(*q*, *t*) = *f*_*α*_(*q*, *t*)*f*_β_(*q*, *t*). This implies that the density relaxation time would be determined by the faster process, namely the α relaxation for *q* < 16 nm^−1^ and the β_JG_ relaxation for *q* > 16 nm^−1^.

**Fig. 3:**
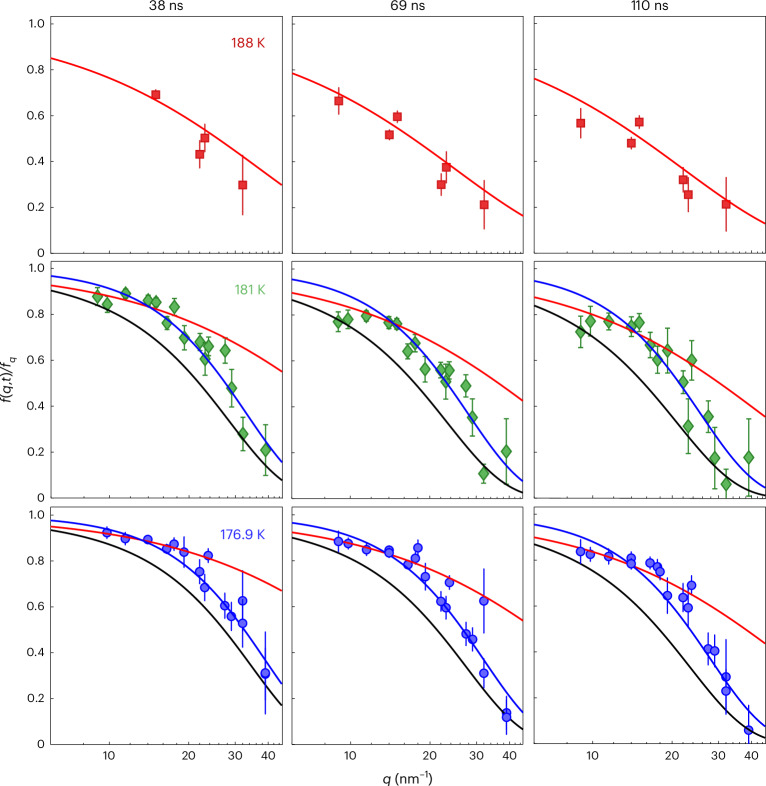
Wavenumber dependence of the intermediate scattering function. Top: the experimental data for *f*(*q*, *t*)/*f*_*q*_ are reported as a function of *q* at three different times (columns) and at three different temperatures (rows) across *T*_αβ_. The full red, blue and black lines correspond to the shape expected for the α relaxation and the β_JG_ relaxation and their product, respectively. The first two contributions describe the data well at low- and high *q*, respectively, but their product fails to describe the overall dataset. At *T* = 188 K > *T*_αβ_ the contribution of the α relaxation is sufficient to describe the *q*-dependence of *f*(*q*, *t*) at each probed time. The error bars correspond to ±1 s.d., obtained after data reduction and logarithmic binning, with uncertainty propagation. The data at 176.9 K combine the results of two independent measuring runs.

To test this hypothesis, we consider the *q*-dependence of the intermediate scattering function *f*(*q*, *t*)/*f*_*q*_ probed at different times, as reported in Fig. 3, for 188, 181 and 176.9 K at three different times.

Following the previous discussion, we model *f*(*q*, *t*) by (1) considering the measured *q*-dependence of the relaxation time and (2) assuming that *f*(*q*, *t*) is well described by a stretched exponential (equation (1)) in the time window that is here probed, with *β*_KWW_ = 0.5 at all *q*. In particular, consistent with the *q*^−2^ dependence of the relaxation time in the α relaxation regime, we can assume for the α relaxation the simple expression

3 $$\frac{{f}_{{\rm{\alpha }}}({\bf{q}},t)}{{f}_{q}^{\,{\rm{\alpha }}}}=\exp \left[-{({D}_{{\rm{\alpha }}}{q}^{2}t)}^{{\beta }_{\mathrm{KWW}}}\right]\approx \exp [-q\sqrt{{D}_{{\rm{\alpha }}}t}],$$

Here ${f}_{q}^{\,{\rm{\alpha }}}$ is the strength of the process, and *D*_α_ is the diffusion coefficient that can be obtained from the quadratic *q*-dependence at high temperatures/small scattering vectors (Fig. 2). Equation (3) is reported in Fig. 3, red lines, and well describes the *f*(*q*, *t*) data at low *q*, in the α relaxation regime. The super-quadratic *q*-dependence of the inverse relaxation time of the β_JG_ relaxation can instead be described in terms of an anomalous diffusion model. By combining the approximately quartic *q*-dependence of the β_JG_ inverse relaxation time with equation (1) for *β*_KWW_ = 0.5, it is possible to write^34,36^

4 $$\frac{{f}_{\mathrm{JG}}({\bf{q}},t)}{{f}_{q}^{\,\mathrm{JG}}}\approx \exp \left[-\widetilde{D}{q}^{2}{t}^{{\beta }_{\mathrm{KWW}}}\right]=\exp \left[-\frac{\langle {r}^{2}(t)\rangle {q}^{2}}{6}\right],$$

where ${f}_{q}^{\,\mathrm{JG}}$ is the relaxation strength of the β_JG_ relaxation. Within this approximation, the normalized intermediate scattering function associated with the β_JG_ relaxation is approximately Gaussian in shape. This allows us to express *f*(*q*, *t*) in equation (4) in terms of the mean-squared molecular displacement, 〈*r*^2^(*t*)〉, which is then related to the anomalous diffusion coefficient $\widetilde{D}$, obtained from fitting the *q*-dependence of the data in Fig. 2, by the relation

5 $$\langle {r}^{2}(t)\rangle =\langle | {\bf{r}}(t)-{\bf{r}}(0){| }^{2}\rangle \simeq 6\widetilde{D}{t}^{{\beta }_{\mathrm{KWW}}}.$$

Equation (4) is reported in all panels of Fig. 3, blue lines, and describes well the *f*(*q*, *t*) data at high *q*, in the β_JG_ relaxation regime.

We are now in the position to test the previously discussed scenario for the α and β_JG_ relaxation processes. The black solid lines reported in Fig. 3 have been calculated as the product of equations (3) and (4), and it is clear that the assumption that the *f*(*q*, *t*) is described by the product of two independent contributions does not match well our experimental data. We also underline that above the crossover temperature (*T*_αβ_ = 181 K), where only the α relaxation is present, equation (3) accounts well for the *q*-dependence of *f*(*q*, *t*) (Fig. 3, top). We can then conclude this section underlining that the transition from diffusive to subdiffusive dynamics seen in the *q*-dependence of the relaxation time is also clearly reflected in the *q*-dependence of the shape of the intermediate scattering function. Around the crossover temperature *T*_αβ_, the α and β_JG_ relaxations cannot be considered as two independent relaxation processes: instead, the relaxation process that affects the density fluctuations changes in nature from what we call the β_JG_ process to the α process as the length-scale (and therefore the timescale) of observation is increased across the Lindemann length. Interestingly, our findings (see also hereafter) provide a microscopic picture of the results of previous NMR investigations establishing a strong correlation between the α and β_JG_ processes^37^.

**Fig. 4:**
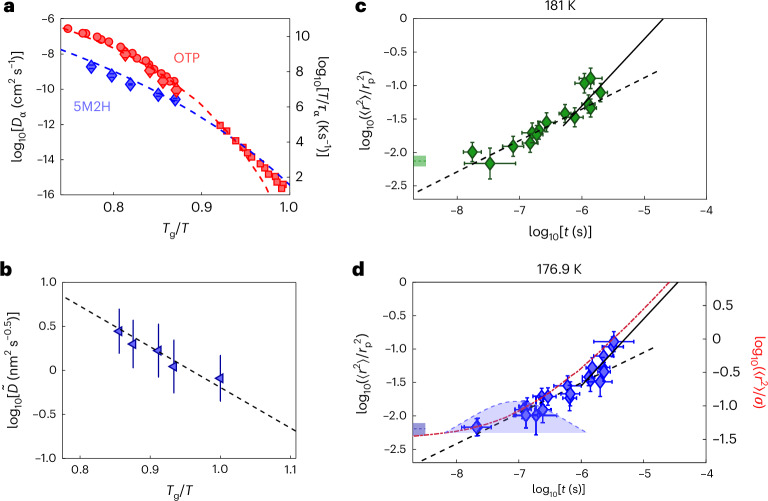
A real-space view of the secondary relaxation in 5M2H. **a**, Left axis: diffusion coefficients extracted from the *q*-dependence of the relaxation time for 5M2H (full blue diamonds, this work) and OTP (full red diamonds, extracted from the data reported in refs. ^12,39^). For OTP, direct measurements of the diffusion coefficient from ref. ^46^ (NMR, red circles) and ref. ^40^ (isothermal desorption, red squares) are reported as well. Right axis: Stokes–Einstein estimation of the temperature dependence of the diffusion coefficient based on literature data for the α relaxation time for 5M2H^29^ (dashed blue line) and OTP^4,41–44^ (dashed red line). **b**, Anomalous diffusion coefficient of 5M2H (full triangles) as a function of the *T*_g_-scaled inverse temperature. The slope of the data is consistent with the expected value of ${E}_{{\rm{JG}}}/(2{k}_{{\rm{B}}}{T}_{{\rm{g}}}{\rm{ln}}(10))$ (dashed line), where *E*_JG_ is the activation energy of the β_JG_ relaxation^29^. **c**, Time dependence of the mean-squared displacement at *T* = 181 K computed from both the α and β_JG_ regimes. The full and dashed lines are the power laws expected in the α and β_JG_ regimes, respectively. **d**, As in **c** but for *T* = 176.9 K. Right axis: the dash-dotted red line refers to numerical simulation data for a three-dimensional size-polydisperse mixture of soft repulsive spheres from ref. ^26^. The blue area in **d** delimited by a dashed line shows the distribution of relaxation times, *G*(log(*τ*)), associated to the β_JG_ relaxation of 5M2H as extracted from dielectric spectroscopy data. The base width correspond to the FWHM of the distribution. The coloured rectangles in **c** and **d** show the mean-squared molecular displacement estimated from the *q*-dependence of the relaxation strength *f*_*q*_ at large scattering vectors (Supplementary Information). The vertical error bars in **a** (some are smaller than the marker size) and **b** and the horizontal error bars in **c** and **d** correspond to ±1 s.d. as obtained from the weighted nonlinear fitting of the experimental data described in the main text. In **c** and **d**, the vertical error bars represent ±1 s.d. as obtained by propagating the uncertainties in the diffusion coefficients ($\widetilde{D}$ and *D*_α_).

The observation that the α-relaxation-controlled regime is characterized by a *q*^−2^ dependence of the relaxation time encourages us to estimate a diffusion coefficient at all probed temperatures, including those where only few *q*-points have been collected. Figure 4a reports the *T*_g_-scaled temperature dependence of the extracted diffusion coefficient (Fig. 4a, blue diamonds, left axis).

Its temperature dependence agrees well with that expected from the Stokes–Einstein equation *D*_α_ ∝ *T*/*τ*_α_ (ref. ^38^) (Fig. 4d, dashed line, right axis), where we have used literature data for the α relaxation time, *τ*_α_ (ref. ^29^). This is interesting, as the diffusion coefficient is known to become *q*-dependent at high *q* (ref. ^38^). We expect that this *q*-dependence should not affect the temperature dependence of *D*_α_, as the *q*-range used to extract it from the experimental data is basically the same at all temperatures. To test this assumption, because we are not aware of other independently measured diffusion coefficient data for 5M2H, we perform the same analysis in the α-dominated regime using literature TDI data for the intermediate scattering function of *o*-terphenyl (OTP) (Fig. 4a, full red diamonds, left axis)^12,39^. OTP has in fact been studied in detail, and direct measurements are available of the diffusion coefficient (Fig. 4a, full red circles and squares, left axis)^40^ and of the timescale of the α relaxation from several techniques^4,41–44^, which we again use to estimate the temperature dependence of *D*_α_ from the Stokes–Einstein expression (Fig. 4a, dashed line, right axis). We note that the same *y*-scales have been used for 5M2H and OTP. We observe that (1) the Stokes–Einstein expression describes the temperature dependence of the diffusion coefficient of OTP well, down to a certain temperature in the deeply supercooled liquid, below which it is violated, as well documented in the literature^45,46^; (2) the direct measurements of the diffusion coefficient in OTP agree rather well (within ~30%) with the estimates obtained from the intermediate scattering function measured at high *q*. We can conclude that it is possible to extract reasonably consistent diffusion coefficients from our data, which gives us confidence in the results of the model used to describe them. Similarly, we can compute the anomalous diffusion coefficient $\widetilde{D}$ associated with the β_JG_ relaxation from the regime where the inverse relaxation time shows an almost quartic dependence on *q* ($\tau ={\widetilde{D}}^{-2}{q}^{-4}$). The data obtained are reported in Fig. 4b as a function of the *T*_g_-scaled inverse temperature. From the relation between $\widetilde{D}$ and the relaxation time of the JG relaxation, which can be worked out from equation (4), we can expect that the slope of ${\log }_{10}(\widetilde{D})$ versus *T*_g_/*T* is ${E}_{{\rm{JG}}}/(2{k}_{{\rm{B}}}{T}_{{\rm{g}}}{\rm{ln}}(10))$, where *E*_JG_ = 0.28 ± 0.02 eV is the activation energy of the β_JG_ relaxation^36^ and *T*_g_ = 155 K is the glass-transition temperature of 5M2H^36^. This prediction is indeed verified in Fig. 4b, sign of an overall internal consistency of the data analysis.

Having worked out and validated a description of our experimental data for the intermediate scattering function at both small and large *q*, we are now in position to extract the time dependence of the mean-squared displacement. In fact, we can associate a time *t* = 1/(*D*_α_*q*^2^) to all *q* where the density fluctuations show a diffusive decay, and we can then evaluate the mean-squared displacement at that time using the simple random-walk relation: 〈*r*^2^(*t*)〉 = 6*D*_α_*t* (ref. ^38^). Similarly, we can associate from equation (4) a time $t=1/({\widetilde{D}}^{2}{q}^{4})$ to all *q* where the density fluctuations are subdiffusive (see also Supplementary Fig. 4 and Supplementary Text 3 for further analysis), and we can then evaluate the mean-squared displacement at that time using the relation: $\langle {r}^{2}(t)\rangle =6\widetilde{D}{t}^{{\beta }_{\mathrm{KWW}}}$. The two regimes cross at ~16 nm^−1^. The values obtained for the mean-squared displacement, after normalization for the intermolecular distance *r*_p_ = 0.76 nm (evaluated from the molecular volume^36^) are reported in Fig. 4c,d for *T* = 181 and 176.9 K, respectively. The shortest time reported here corresponds to the highest *q* that we have reached, whereas the longest time corresponds to the shortest investigated *q*. Moreover, we also report in Fig. 4c,d the mean-squared displacement (coloured rectangles), computed from the *q*-dependence of the relaxation strength *f*_*q*_ (see Supplementary Fig. 3 for details). The full and dashed lines in Fig. 4c,d are the power laws expected in the α (〈*r*^2^(*t*)〉 ∝ *t*) and β_JG_ (〈*r*^2^(*t*)〉 ∝ *t*^0.5^) regimes, respectively, and are well consistent with the data. It is remarkable that the mean-squared displacements derived from two different regimes in *q* display such a continuous time dependence.

At both temperatures, our data correspond to the portion of the time dependence of the mean-squared displacement comprised between the exit from the plateau (which we barely reach) and the start of the diffusion regime. They clearly show that, although the α relaxation corresponds to the diffusion regime, as already known and expected, the β_JG_ relaxation corresponds to a sublinear regime in the mean-squared displacement that follows the plateau and precedes the diffusion regime. In Fig. 4d, we also report the distribution of relaxation times associated with the β_JG_ process (blue area) as extracted from dielectric measurements^29^. The overlap in time between this distribution and the sublinear regime that appears in the mean-squared displacement is noticeable. Therefore, in 5M2H, the β_JG_ relaxation corresponds to the critical rattling of the molecules within the cage of their closest neighbours just before molecular diffusion, borrowing from the classical mode-coupling theory a terminology used for the von Schweidler’s regime predicted above the critical temperature *T*_c_ (ref. ^47^). We underline that the sublinear time dependence of the mean-squared displacement reported in Fig. 4 covers a wide range of two decades and more in time and reflects the width of the distribution of relaxation times for the β_JG_ relaxation: in the framework presented here, it is the *q*-dependence of the β_JG_ relaxation that translates a relaxation peak (as observed, for example, in dielectric spectroscopy) into a power law in time.

In Fig. 4d (right axis) we also report the simulation data for a three-dimensional size-polydisperse mixture of soft repulsive spheres from ref. ^26^ (dash-dotted red line, right axis). This dataset was selected because it corresponds to the same value of *τ*_α_ (in physical units) as in our data. Specifically, we selected the mean-squared displacement at *T* = 0.085 (in reduced units), for which *τ*_α_ ≈ 2 μs (one simulation unit ~3 × 10^−11^ s (ref. ^26^)). The consistency between the two datasets is remarkable. Although these simulation data have not been analysed similarly to what we do here, the match between the experimental and simulation data shown in Fig. 4d opens the possibility that the conclusions that we have reported here for 5M2H might be valid for a broad class of systems.

Although from the available data for 5M2H we cannot accurately estimate the location of the mode-coupling temperature, *T*_c_, it has often been reported that *T*_c_ ≈ *T*_αβ_ ≈ 1.2*T*_g_ (ref. ^48^). This suggests that the sublinear diffusion process that we report here below *T*_αβ_ might also be related to *T*_c_. It is then interesting to observe that recent molecular dynamics simulations have revealed the emergence across *T*_c_ of a novel subdiffusive regime, responding to a different underlying mechanism than above *T*_c_ (ref. ^49^), which might be related to the subdiffusive regime that we discuss here. This is a critical time and temperature range to advance our understanding of the mechanisms that lead to the glass transition. For instance, the onset of subdiffusion has recently been related to emergent facilitation and correlated dynamics in a two-dimensional lattice model of glassy behaviour^50^, and facilitation is a mechanism that is becoming more and more the focus of recent investigations^7,26^. Combining these results with our observations, we can infer that the β_JG_ process might be related to facilitation, a fascinating connection that will require more investigation.

In conclusion, here we propose an experimentally extracted real-space picture of the β_JG_ relaxation in the temperature range where it decouples from the structural process. Our results show that the α and β_JG_ relaxations cannot be treated as two independent processes: the microscopic mechanism affecting density fluctuations evolves from what we call the β_JG_ process to the α process as the length-scale (and therefore the timescale) of observation is increased. The β_JG_ relaxation can be associated with the subdiffusive regime preceding the onset of the α relaxation but not independent of it and possibly inducing or facilitating it. We also stress that, although the results presented here refer to a typical hydrogen-bonded glass former, 5M2H—the sample where we first managed to present a detailed study in the time and temperature range where the α and β_JG_ relaxations decouple—they are consistent with partial results available in the literature for different classes of glass formers^12,30,36,51^ and are probably generic. We are, however, aware that still much has to be done to solve the many remaining inconsistencies. For instance, long simulations using the Kob–Andersen Lennard–Jones mixture, which is surely the most used model to study the glass transition, do not seem to show the transition in *q* between the α and the β_JG_ relaxation that we report here^52^, though a qualitative change in the subdiffusive dynamics across *T*_c_ is observed^49^. We might advocate that this model refers to a rather strong glass former, for which the β_JG_ relaxation is known to be weak or rather merged with the α relaxation^53,54^, though this issue requires further investigation. Moreover, recent numerical simulations^23^ of an asymmetric dimer system report that the self intermediate scattering function displays both the α and β_JG_ relaxations even above *T*_c_, unlike our results, which show that these two processes correspond to two different *q*-ranges (length scales). Recent depolarized light-scattering experiments^55^ and a large corpus of dielectric spectroscopy data^20^, which, however, refer to a different observable, also report evidence of both processes in the same spectrum. The distinction between universal and system-specific effects, as well as the comparison of results across different observables, remains a challenge which will require sustained, collective efforts from the scientific community studying the glass-transition. We believe that the results reported here contribute to this effort by clarifying the role of the β_JG_ process as a precursor of the α relaxation and thus its role in the glass transition.

## Methods

### Sample

We purchased 5M2H (*T*_g_ = 155 K (ref. ^36^)) from Sigma-Aldrich (purity ~98%) and used it without further purification. It exhibits a well-resolved β_JG_ relaxation process in dielectric spectroscopy, temporally decoupled from the primary structural relaxation above *T*_g_, and thus is well suited for investigations within the timescale accessible to the TDI experiment described hereafter. We studied 5M2H here in its supercooled liquid state using a He-flow cryostat that controlled the temperature with stability ±0.05 K.

The intermolecular nature of the β_JG_ process in 5M2H has already been established in previous work^28^. In addition to the α and β_JG_ relaxations, the dielectric spectra of monohydroxyl alcohols exhibit the so-called Debye peak. This feature is intrinsic to the dielectric response and is generally absent in other response functions, such as in density fluctuations^29^ or in the orientational susceptibility measured by photon-correlation spectroscopy^58^. Importantly, the activation barrier and the characteristic timescale of the α relaxation obtained from dielectric spectroscopy (where it is partially obscured by the Debye peak) and from photon-correlation spectroscopy (where the Debye contribution is absent) are found to coincide^58^. We can therefore conclude that the relaxation spectrum of 5M2H is well established.

### Time-domain interferometry

In a nuclear γ-resonance time-domain interferometry experiment, nuclear resonant scattering of X-ray radiation from two nuclear absorbers, containing the Mössbauer isotope ^57^Fe and placed upstream and downstream of the investigated sample, is used to produce a time-evolving quantum beat^56,57,59^. The nuclear resonance of ^57^Fe lies at an energy of approximately 14.4 keV, which can be easily reached by using synchrotron radiation. It has a lifetime *τ*_0_ ≈ 141 ns (corresponding to a natural line width *Γ*_0_ ≈ 4.66 neV) that matches the timescale of the β_JG_ relaxation of 5M2H near the glass-transition temperature *T*_g_. The contrast of the TDI beating pattern is modulated by the microscopic density fluctuations *ρ*(**q**, *t*) of the sample, and proper data processing allows the extraction of the intermediate scattering function

6 $$f({\bf{q}},t)=\frac{\left\langle \rho ({\bf{q}},0)\rho ({\bf{q}},t)\right\rangle }{\left\langle | \rho ({\bf{q}},0){| }^{2}\right\rangle },$$

where *ρ*(**q**, *t*) is the *q*-component of the microscopic density at time *t* (see Supplementary Information for more details).

The present experiment was carried out on the ID18 nuclear resonance beamline of the European Synchrotron Radiation Facility (ESRF)^60^ with the storage ring operating in the four-bunch filling-mode (storage ring current *I* = 20 mA). In such an operation mode, the time interval between two synchrotron radiation pulses is 704 ns. The incident X-ray radiation was characterized by a bandwidth of 2.3 meV at the energy of the first nuclear transition of ^57^Fe (14.412 keV). The bandwidth was selected using a high heat-load and a high resolution monochromator in cascade.

A three-line TDI setup was used consisting of an α-^57^Fe foil (thickness 10 μm) that generates the two-line probe beam, whereas the reference single-line absorber consisted of a pellet containing K_2_Mg^57^Fe[CN]_6_ (^57^Fe surface density of 1 mg cm^−^^2^). To reduce the number of nuclear resonances of α-^57^Fe to two, we applied a magnetic field of 0.5 T perpendicularly to the horizontal scattering plane (see refs. ^57,61^ for more details). This scheme was chosen because multi-line TDI experiments are more efficient compared with standard two-line schemes and allow us to directly extract the relaxation strength of the probed process^51,56,57^.

The TDI signal was collected simultaneously at three different scattering vectors (*q*) to reduce the total time of the experiment. In detail, we used three different groups of avalanche photodiode detectors (time resolution of ≃1 ns), each of them probing a different scattering angle. By changing the position of the detectors with respect to the sample we could select the *q*-values of interest. The absence of crystallization during data acquisition was monitored by measuring the static structure factor of 5-methyl-2-hexanol at the end of every isothermal acquisition.

## Online content

Any methods, additional references, Nature Portfolio reporting summaries, source data, extended data, supplementary information, acknowledgements, peer review information; details of author contributions and competing interests; and statements of data and code availability are available at 10.1038/s41567-026-03320-5.

## Supplementary information

Supplementary Information Supplementary Figs. 1–4, Texts 1–3 and Table 1.

Peer Review File

## Data Availability

All data used to generate Figs. 1–4 are available via figshare at 10.6084/m9.figshare.31814242 (ref. ^62^).
