# Supplementary material for: Crossover of quasi-localized dynamics and diffusion in supercooled liquids
Source: Nat Phys. 2026 Jun 9;22(8):1298–305. doi: 10.1038/s41567-026-03320-5 (PMC13461351; doi:10.1038/s41567-026-03320-5)
Supplement: Supplementary file 1 [file 41567_2026_3320_MOESM1_ESM.pdf]

nature physics

Supplementary information https://doi.org/10.1038/s41567-026-03320-5

# Crossover of quasi-localized dynamics and diffusion in supercooled liquids

In the format provided by the authors and unedited

**TABLE OF CONTENT**


- S1 Analysis of TDI interferograms and extraction of the intermidiate scattering function.
- S2 Fixing the stretching exponent $\beta_{KWW}$
- S3 Relaxation strength and mean-squared displacement
- Supplementary figure S1-S4

## I. ANALYSIS OF TDI INTERFEROGRAMS AND EXTRACTION OF THE INTERMIDIATE SCATTERING FUNCTION.

In a Time-Domain Interferometry (TDI) experiment, the first nuclear absorber acts as a split and delay line: a $\gamma$-photon impinging onto it can be transmitted or resonantly absorbed by the long-lived nuclear resonance of $^{57}$Fe. The second absorber, given its different excitation spectrum, behaves as a phase-sensitive analyzer: when the scattering paths recombine at the downstream absorber, a time-domain pattern of quantum beats arises, with a period and structure fully determined by the energy spectra of the two absorbers. In the present experiment, the interferogram consists of two groups of quantum beats with frequency $\Omega \simeq 60\Gamma_0$ and $\Omega/2 \simeq 30\Gamma_0$, respectively, where $\Gamma_0 \approx 4.66\,\mathrm{neV}$ is the natural linewidth of the nuclear resonance [1]. The coherent superposition of the paths is mediated by the quasi-elastic scattering from the sample placed in between the two absorbers. In general, microscopic relaxations in the sample lead to a loss of phasing and a consequent damping of the beating pattern [2–4]. In the specific case of the 3-line scheme used here, quasi-elastic scattering reduces the contrast between the beatings with frequencies $\Omega$ and $\Omega/2$ (see, for example, Fig. S1).

The time evolution of the beating pattern can be quantitatively described using, for the intensity, $\bar{I}(\mathbf{q},t)$, measured for a scattering vector $q$ and at time $t$, the model introduced in Refs. [1, 4]:

$$\begin{aligned}\bar{I}(\mathbf{q},t) &= |R_A(t)|^2 + |R_B(t)|^2 \cdot f_{\Delta E}(\mathbf{q}) \\ &+2Re\{R_A(t,T)\cdot R_B(t,T)\} f(\mathbf{q},t)\,.\end{aligned} \tag{1}$$

In Eq. 1 $R_{A,B}(t)$ are the responses of the probe (two-line, A) and reference (single-line, B) absorber, respectively; $f(\mathbf{q},t)$ is the normalized density-density correlation function or intermediate scattering function:

$$f(\mathbf{q},t) = \frac{\langle \rho(\mathbf{q},0)\rho(\mathbf{q},t)\rangle}{\langle |\rho(\mathbf{q},0)|^2\rangle}; \tag{2}$$

and $f_{\Delta E}$ is a parameter depending both on sample properties and on the bandwidth of the incident radiation [3]. More in detail, $f_{\Delta E}$ can be written as:

$$f_{\Delta E} = \frac{\int_{-\infty}^{\infty} S(\mathbf{q},\omega) I(-\omega) d\omega}{\int_{-\infty}^{\infty} S(\mathbf{q},\omega) d\omega}\,. \tag{3}$$

In Eq. 3, $S(\mathbf{q},\omega)$ is the dynamic structure factor and $I(-\omega)$ is the area-normalized energy spectrum of the incident SR pulse. In presence of a plateau in the intermediate scattering function of the system, $f_{\Delta E}$(q) can be regarded as the value of the intermediate scattering function $f(\mathbf{q})$

evaluated at $\Delta T \approx 2\hbar/\Delta E$, where $\Delta E$ is the bandwidth of the incident synchrotron radiation pulse [4]. In our experimental conditions $\Delta E \approx 2.5$ meV, which implies that $f_{\Delta E} \approx f(q, t = 0.5 \text{ ps})$. A detailed description of the models used to describe $R_{A,B}(t)$ and the extraction of $\Delta E$ is reported in [1, 4]. $R_{A,B}(t)$ can be calibrated in absence of the sample or, as in the present case, from interferograms measured at temperatures (25 K) much lower than $T_g$, where the sample is expected to show no dynamics in the probed time-window. This approach also allows us to estimate possible instrumental losses of coherence. $f_{\Delta E}$ and $f(\mathbf{q}, t)$ are extracted by a fitting procedure to the beating patterns. It is important to note that $f_{\Delta E}$ and $f(\mathbf{q}, t)$ can be separated in the fitting procedure only

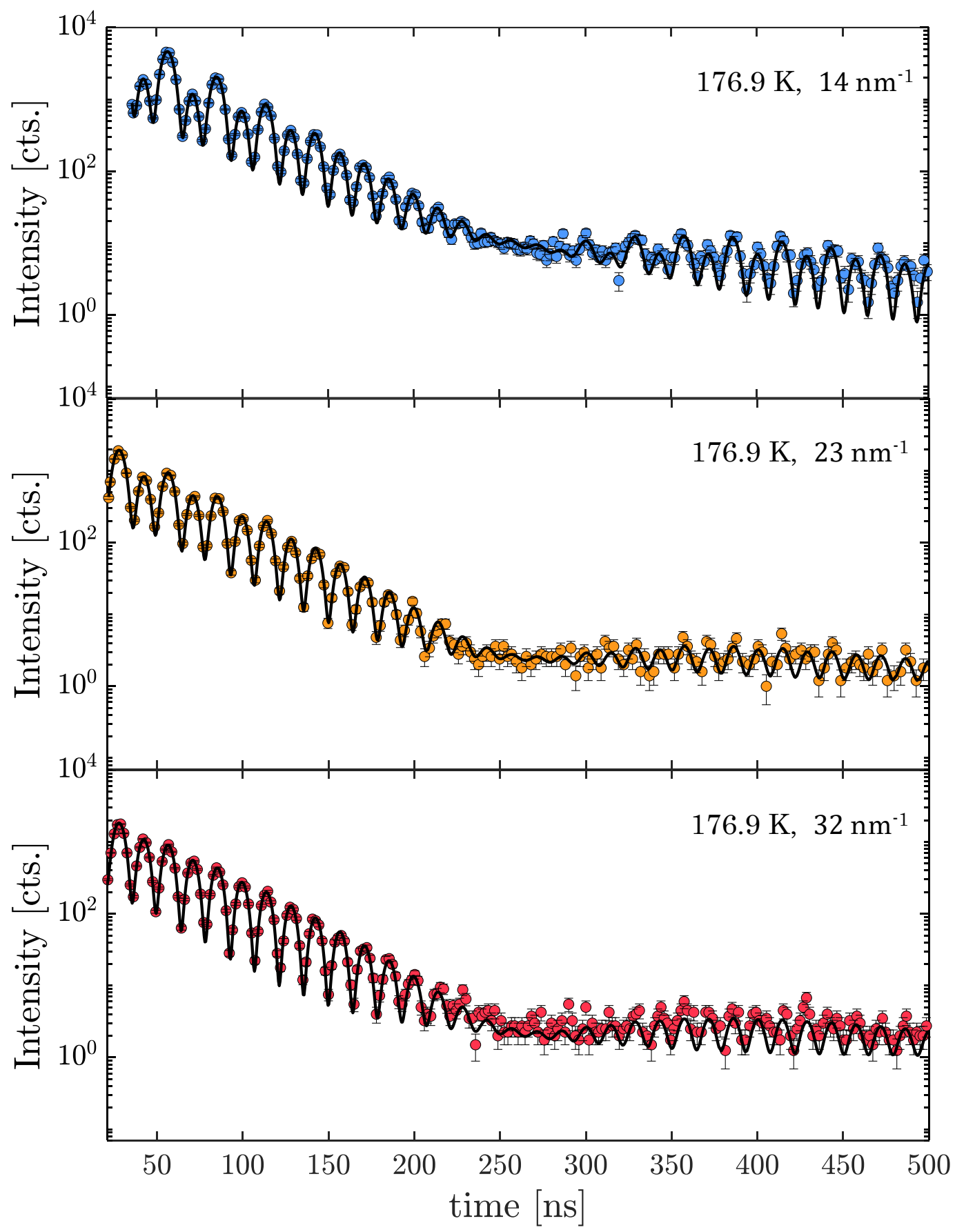


FIG. S1. Time-domain interferometry beating patterns of 5-methyl-2-hexanol at T= 176.9 K at different scattering vectors. The symbols with errorbars are the experimental data, while the black-solid lines are the model curves obtained from the fitting procedure. To increase visibility, the raw data have been averaged over ±0.9 ns. Errorbars denote ±1 S.D., computed assuming Poisson statistics by summing the counts within each bin and propagating the resulting uncertainty. The extracted intermediate scattering functions are reported in Fig. 1(A) of the main text.

when the intrumental responses of the probe $|R_A(t)|$ and reference $|R_B(t)|$ absorber are different [3]. This condition is naturally achieved in a 3-line TDI scheme [4].

The intermediate scattering function is usually described by the KWW function [5] as it provides a good account of the experimental data [6, 7]:

$$f(\mathbf{q},t) = f_q \exp\left[-\left(\frac{t}{\tau(\mathbf{q},t)}\right)^{\beta_{KWW}}\right]. \tag{4}$$

In Eq. 4, $f_q$ is the relaxation strength (i.e. the fraction of molecules participating in the relaxation process), $\tau(\mathbf{q})$ the relaxation time of the density fluctuations, and $\beta_{KWW}$ is the stretching parameter. Although the $\alpha$- and the $\beta_{JG}$-relaxations show different spectral shapes in the dielectric spectra [5], here using the single shape of Eq. 4 independently of the details of the underlying dynamical process is preferred as it introduces a minimal bias in the fitting procedure. Furthermore, as also discussed in the methods section of Ref. [7], TDI beating patterns span less than two decades in time and are not very sensitive to the long-times, stretched tails characteristic of the susceptibility in the time-domain. For the same reason, the relaxation parameters are not critically sensitive to the exact shape of the model selected to describe the intermediate scattering function, as discussed in the following section. Examples of TDI interferograms along with the fitting curves obtained using Eq. 1 are reported in Fig. S1.

## II. FIXING THE STRETCHING EXPONENT $\beta_{KWW}$

We find, by jointly fitting the data measured at several temperatures and scattering vectors, that $\beta_{KWW} = 0.44 \pm 0.08$. In detail, we jointly fitted data at different temperatures and scattering vectors where $\beta_{JG}$- or $\alpha$-relaxation is active. The values obtained for $\beta_{KWW}$ are reported in Tab. I. The average value that we have here obtained is compatible with the value used in previous work on 5-methyl-2-hexanol, i.e. $\beta_{KWW} = 0.5$ [7]. This value has then been fixed in the rest of our analysis and further validated as discussed below. It is important to stress that our results are unaffected by variations of $\beta_{KWW} \pm 0.2$ and that the extraction procedure of the scattering function is not influenced by a specific choice of $\beta_{KWW}$ in that range, see e.g. Fig. S2.

TABLE I. Stretching parameter $\beta_{KWW}$ extracted from globally-fitted time-domain interferometry beating patterns in different temperature and wave-number ranges. The uncertainties are $\pm 1$ S.D..

| $q$-range [$\mathrm{nm}^{-1}$] | $T$-range [K] | $\beta_{KWW}$ // |
|---|---|---|
| 32 | 155-177 | $0.5 \pm 0.3$ |
| 22-39 | 177, 181 | $0.42 \pm 0.13$ |
| 22-39 | 155-200 | $0.5 \pm 0.2$ |
| 14 | 155-200 | $0.41 \pm 0.13$ |

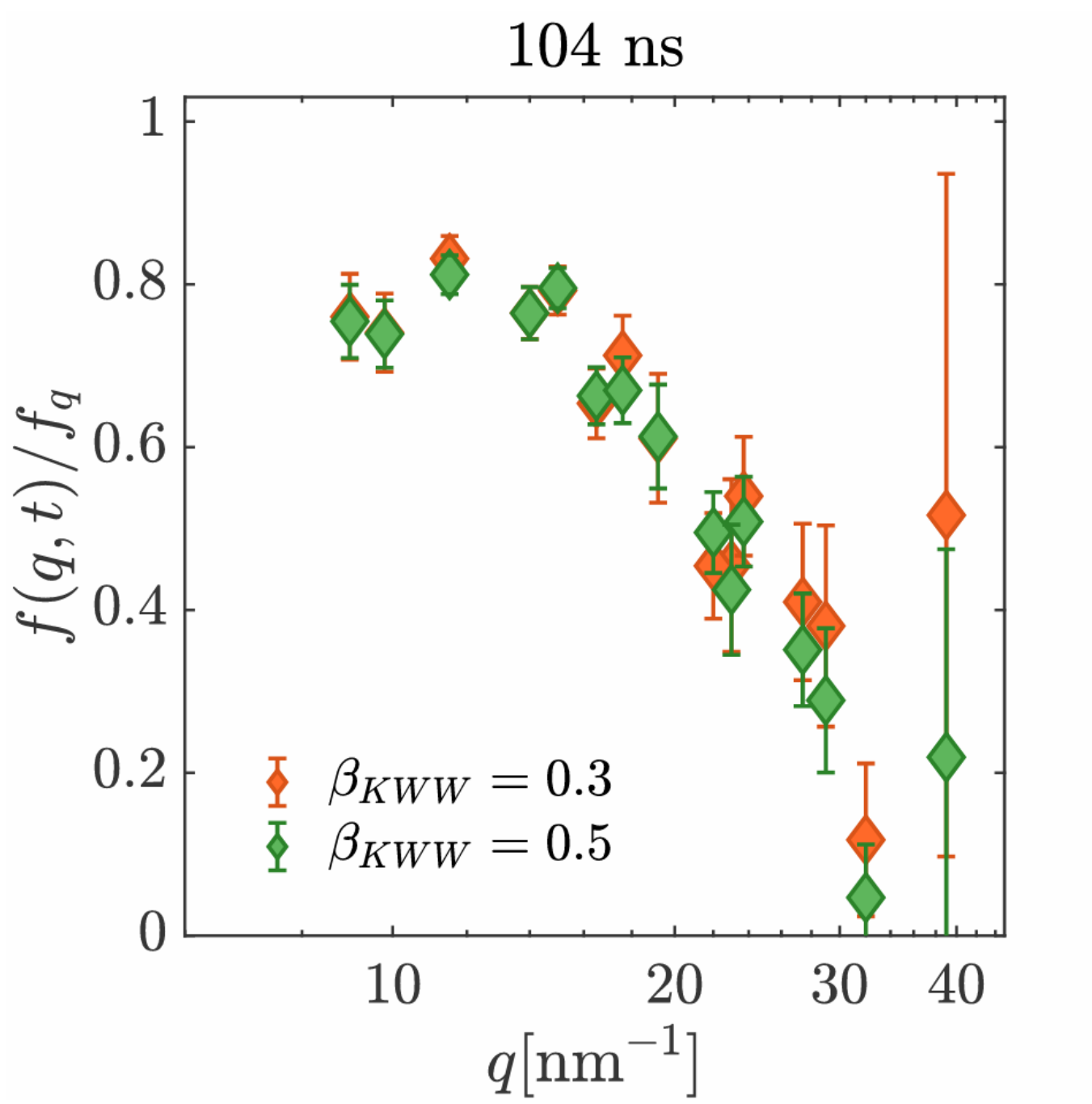


FIG. S2. Wavenumber dependence of the re-normalized intermediate scattering function evaluated at 104 ns extracted using two different values of $\beta_{\mathrm{KWW}}$, namely 0.3 (orange diamonds) and 0.5 (green diamonds). The specific choice of $\beta_{\mathrm{KWW}}$ does not influence critically the extraction of the intermediate scattering function from the measured interferograms. Error bars denote $\pm 1$ S.D., computed after weighted fitting and reduction of the TDI beating patterns as discussed in Fig. 1.(A). The S.D. combines the within-bin variance with propagated uncertainties from the data-reduction procedure and the normalization by $f_q$.

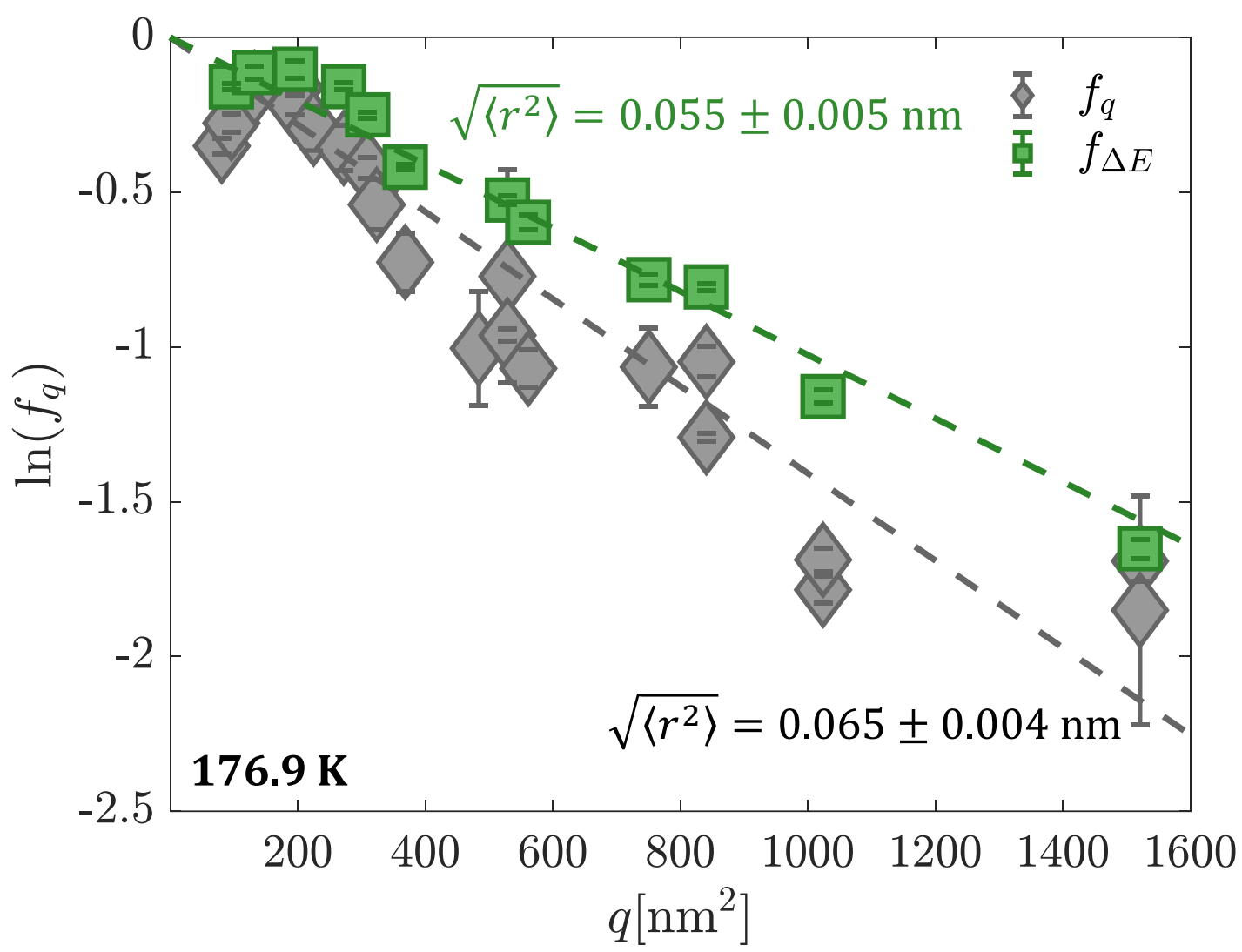


FIG. S3. Natural logarithm of the relaxation strengths, $\ln f_q$ (gray diamonds) and $\ln f_{\Delta E}$ (green squares), as a function of the square of the exchanged wave number, $q^2$, at 176.9 K. The dashed lines are linear fits to the data. The errorbars show $\pm 1$ S.D, obtained from the weighted non-linear fitting of the time-domain beating patterns.

## III. RELAXATION STRENGTH AND MEAN-SQUARED DISPLACEMENT

The relaxation strength, $f_q(T)$, extracted fitting the KWW function to the intermediate scattering function data, is reported Fig. S3. The data show a broad peak in correspondence to the first peak in the scattered intensity reported in Fig. 1(B) in the main text, as typically observed in liquids [8]. At higher $q$'s, the $f_q(T)$ data decay with $q$. In this high-$q$ range, the structure factor, $S(q)$, oscillates around one displaying weaker and weaker oscillations on increasing $q$, and it becomes more and more appropriate to describe the intermediate scattering function neglecting the atomic correlations, i.e. approximating it with its self-component [9]. Additionally invoking the harmonic approximation to describe the atomic vibrations around the "instantaneous" positions (before relaxation) [9], we can write:

$$f_q = e^{-2W} = \exp\left[-\frac{q^2 \langle r^2(t) \rangle}{3}\right] \tag{5}$$

where $\langle r^2(t) \rangle$ is the mean squared molecular displacement (MSD) used in the main text. It is therefore possible to fit Eq. 5 to the data for $f_q$ at high $q$ (we use here $q > 16\mathrm{nm}^{-1}$) to extract the MSD at the corresponding temperature, see the dashed lines weighted fitting the $f_q$ and $f_{\Delta E}$ data in Fig. S3. Following the approximations used to derive them, they can be associated to a time

preceding the start of the relaxation process and comprised between the shortest time accessed in the experiment (∼10 ns) and 0.5 ps, with the latter value corresponding to the characteristic timescale associated to $f_{\Delta E}$. The averages of the so-obtained values are plotted in Fig. 4(C) and (D) as horizontal bands at short times.

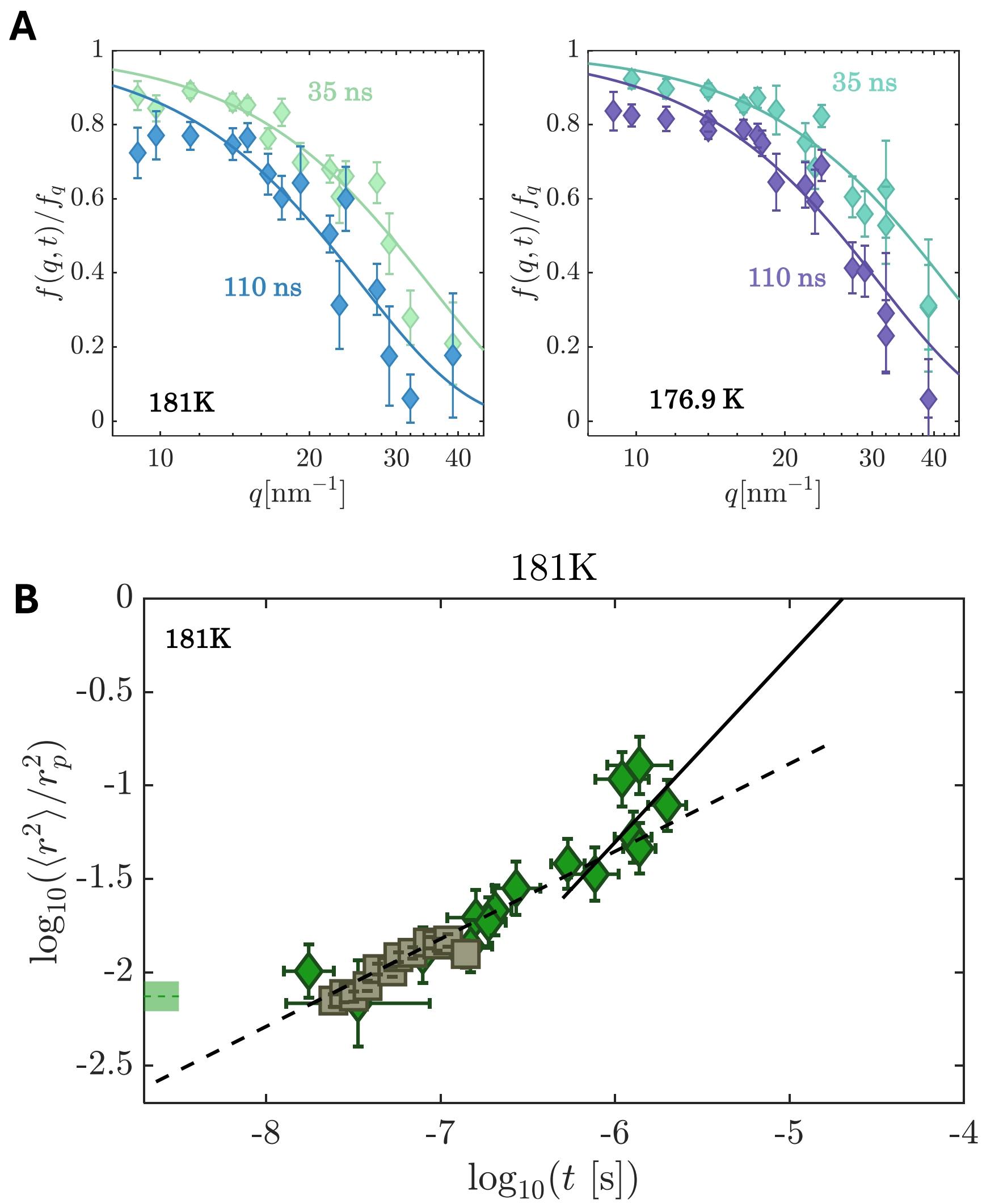


FIG. S4. A: Renormalized intermediate scattering function $f(q,t)/f_q$ as a function of $q$ at two different times (35 ns and 110 ns) and at 181 K and 176.9 K. The solid lines are Gaussian fits (see Eq. 4 in the main text). The uncertainties correspond to ±1 S.D., as discussed in Fig.1-(A). B: Green diamonds: time-dependence of the mean-squared displacement at $T$ =181 K computed from both the $\alpha$ and $\beta_{JG}$ regimes as in Fig. 4 of the main text (see there for details on the reported uncertainties). Gray squares: mean-squared displacement extracted from weighted fitting the $f(q,t)/f_q$ data using Eq. 4 in the main text. The errorbars correspond to ±1 S.D. resulting from the Gaussian fit of the q-profiles.

## DIRECT ANALYSIS OF THE $q$-DEPENDENCE OF THE INTERMEDIATE SCATTERING FUNCTION

The renormalized intermediate scattering function at short times is expected to be dominated by the $\beta_{JG}$-relaxation and to exhibit a Gaussian profile, as discussed in the main text (see Eq. 4). By fitting its exchanged wave-vector ($q$) dependence with a Gaussian function, one can directly extract the corresponding mean-squared displacement.

Fig. S4(A) shows the fits to the re-normalized intermediate scattering functions measured at 181 K for two different delay times, 35 ns and 110 ns, using Eq. 4 in the main text. The data are clearly compatible with a Gaussian profile. The resulting mean-squared displacements are presented in Fig. S4(B) (gray squares), together with those extracted from the $q$-dependence of the relaxation time (green diamonds) and discussed in the main text. The excellent agreement between the two sets of data from these two independent analysis procedures provides an additional consistency check of our results.

---


[1] F. Caporaletti, A. Chumakov, R. Rüffer, and G. Monaco, Review of Scientific Instruments **88**, 105114 (2017).

[2] A. Baron, H. Franz, A. Meyer, R. Rüffer, A. Chumakov, E. Burkel, and W. Petry, Physical review letters **79**, 2823 (1997).

[3] M. Saito, R. Masuda, Y. Yoda, and M. Seto, Scientific reports **7**, 1 (2017).

[4] F. Caporaletti, A. I. Chumakov, R. Rüffer, and G. Monaco, Philosophical Magazine **100**, 2646 (2020).

[5] K. Ngai, *Relaxation and Diffusion in Complex Systems* (Springer New York, 2011).

[6] M. Saito, S. Kitao, Y. Kobayashi, M. Kurokuzu, Y. Yoda, and M. Seto, Physical Review Letters **109** (2012).

[7] F. Caporaletti, S. Capaccioli, S. Valenti, M. Mikolasek, A. I. Chumakov, and G. Monaco, Nature Communications **12** (2021).

[8] in *Theory of Simple Liquids (Fourth Edition)*, edited by J.-P. Hansen and I. R. McDonald (Academic Press, Oxford, 2013) fourth edition ed., p. i.

[9] N. Berk, Journal of Research of the National Institute of Standards and Technology **98**, 15 (1993).
