# Supplementary material for: Crossover of quasi-localized dynamics and diffusion in supercooled liquids
Source: Nat Phys. 2026 Jun 9;22(8):1298–305. doi: 10.1038/s41567-026-03320-5 (PMC13461351; doi:10.1038/s41567-026-03320-5)
Supplement: Supplementary file 2 [file 41567_2026_3320_MOESM2_ESM.pdf]

nature portfolio

Peer Review File

Crossover of quasi-localized dynamics and diffusion in supercooled liquids

Corresponding Author: Dr Federico Caporaletti

Version 0:

Reviewer comments:

Reviewer #1

(Remarks to the Author)
This manuscript presents a very interesting study on the relation between the alpha relaxation and the Johari Goldstein relaxation that comes from a well known group. The authors frame the discussion in a general context and then perform the measurements on a specific molecule, a typical glass former, the 5-methyl-2-hexanol, in its liquid supercooled state down to low supercooled temparatures. They use a wavevector-resolved x-ray technique to access microscopic, real-space information. The results are very interesting and certainly worth a nature physics but the interpretation would benefit from more discussion.
Here are the points that I'd like the authors to better discuss.
1) The alpha relaxation is a translational relaxation time. Does their spectroscopy measure only translations? Because they compare their results with dielectric relaxation that is a different cross section and measures rotations. I ask the authors to explain to the readers what quantities different cross sections measure, when comparing them.
2) Can the Johari Goldstein relaxation be associated to rotations, or do the authors think that is a translational process, or do they have no clue?
3) In water, a supercooled molecular liquid that follows Mode Coupling Theory, rotations and translations are coupled while molecules rattle in the cage and when the cage relaxes they decouple. See for example Chen et al, PRE 56, 4231 (1997) where this phenomenon is clearly shown. Do their conclusions on 5-methyl-2-hexanol support this picture?

Reviewer #2

(Remarks to the Author)
The authors use nuclear gamma-resonance time-domain spectroscopy to gain insight into comparing the alpha and JG-beta relaxations in a hydrogen-bonded liquid, 5M2H. The data clearly reveal a change in temperature dependence at Tc, but more importantly a change in the q-dependence from diffusive to sub-diffusive motion, indicating that the beta-relaxation is the cage-rattling process, beyond which diffusion sets in.
The data support the conclusions, but similar works by the same authors have arrived at the same overall conclusions [their ref. 19, 30, 35]. The present study is more detailed than previous ones, but lacks novelty regarding the conclusions. Also, previous work from other authors have indicated this nature of the beta-relaxation observed here.
The paper and data presentation are very clear, with appropriate references. However, the clarity could be improved by adding discussions regarding the effect of 5M2H being a mono-alcohol, where often another slower peak is seen in BDS data, possibly adding uncertainty regarding the interpretation of the main relaxation peak.
Overall, this work shows improved results concerning an interesting topic that deserves publication, but lacking the novelty required for Nature Physics.

Version 1:

Reviewer comments:

Reviewer #1

(Remarks to the Author)
The authors have replied to all the points that I raised satisfactorily and the current version of the paper is now further improved. For me it can be accepted in the present form.

Reviewer #2

(Remarks to the Author)
With the additional explanations in the response letter and the changes in the text, I feel the authors have acted convincingly on the criticism of both reviewers. In its revised form, the paper is now recommended for publication in Nature Physics.

Open Access This Peer Review File is licensed under a Creative Commons Attribution 4.0 International License, which permits use, sharing, adaptation, distribution and reproduction in any medium or format, as long as you give appropriate credit to the original author(s) and the source, provide a link to the Creative Commons license, and indicate if changes were made.
In cases where reviewers are anonymous, credit should be given to 'Anonymous Referee' and the source.
The images or other third party material in this Peer Review File are included in the article's Creative Commons license, unless indicated otherwise in a credit line to the material. If material is not included in the article's Creative Commons license and your intended use is not permitted by statutory regulation or exceeds the permitted use, you will need to obtain permission directly from the copyright holder.
To view a copy of this license, visit https://creativecommons.org/licenses/by/4.0/

Reviewer 1

This manuscript presents a very interesting study on the relation between the alpha relaxation and the Johari Goldstein relaxation that comes from a well known group. The authors frame the discussion in a general context and then perform the measurements on a specific molecule, a typical glass former, the 5-methyl-2-hexanol, in its liquid supercooled state down to low supercooled temperatures. They use a wavevector-resolved x-ray technique to access microscopic, real-space information. The results are very interesting and certainly worth a Nature Physics but the interpretation would benefit from more discussion.

Reply: We thank the Reviewer for deeming our results interesting and worth publication in Nature Physics. In the following we address her/his concerns and comments point-by-point.

1) The alpha relaxation is a translational relaxation time. Does their spectroscopy measure only translations? Because they compare their results with dielectric relaxation that is a different cross section and measures rotations. I ask the authors to explain to the readers what quantities different cross sections measure, when comparing them.

   Reply: We thank the Reviewer for rising this point. Dielectric spectroscopy mainly probes molecular rotations, via electric dipole fluctuations; X-ray quasi-elastic scattering probes density fluctuations, as discussed in the Methods section, and can in principle be sensitive to both molecular translations and rotations. While we are not in position to disentangle these two contributions in our data, we observe that, depending on the probed wavenumber, density fluctuations couple to either the structural relaxation (at low q) or to the Johari-Goldstein relaxation (at high q). These processes are also observed in dielectric spectroscopy studies, from which we can conclude that electric dipole fluctuations and density fluctuations are coupled at around Tc in the sample studied here. A comment along these lines has now been added to the introduction of our manuscript.

2) Can the Johari Goldstein relaxation be associated to rotations, or do the authors think that is a translational process, or do they have no clue?

   Reply: The Johari Goldstein relaxation is surely associated to rotations, given its visibility in dielectric spectroscopy spectra. From our data and literature data we can conclude that the Johari-Goldstein relaxation is also coupled to both electric dipole and density fluctuations at high q. Most likely, at the relatively low temperatures explored here, rotations and translations are coupled, though a detailed MD simulation would be required to specifically address this point.

3) In water, a supercooled molecular liquid that follows Mode Coupling Theory, rotations and translations are coupled while molecules rattle in the cage and when the cage relaxes they decouple. See for example Chen et al, PRE 56, 4231 (1997) where this phenomenon is clearly shown. Do their conclusions on 5-methyl-2-hexanol support this picture?

   Reply: We thank the Reviewer for the interesting reference. As mentioned above, our measurements directly indicate that electric dipole fluctuations and density fluctuations are coupled both during molecular rattling in the cage (Johari-Goldstein relaxation) and during structural relaxation. In particular, we observe that density fluctuations couple to the Johari-Goldstein at high q where, according to the cited paper, roto-translational coupling is strong. Actually, the temperature range explored here (across Tc) is lower than that explored in the mentioned simulation work, so roto-translational coupling might be even stronger than in that case. Therefore,

it is possible that the Johari-Goldstein relaxation is mainly associated to rotations and that it is the roto-translational coupling, which is small at low-q, the mechanism that makes the Johari-Goldstein relaxation visible in the density fluctuations at high q, consistently with the mentioned simulation work. We added a comment along those lines in the results/discussion section of our manuscript.

Reviewer 2

The authors use nuclear gamma-resonance time-domain spectroscopy to gain insight into comparing the alpha and JG-beta relaxations in a hydrogen-bonded liquid, 5M2H. The data clearly reveal a change in temperature dependence at Tc, but more importantly a change in the q-dependence from diffusive to sub-diffusive motion, indicating that the beta-relaxation is the cage-rattling process, beyond which diffusion sets in. The data support the conclusions, but similar works by the same authors have arrived at the same overall conclusions [their ref. 19, 30, 35]. The present study is more detailed than previous ones, but lacks novelty regarding the conclusions. Also, previous work from other authors have indicated this nature of the beta-relaxation observed here. The paper and data presentation are very clear, with appropriate references.

Reply: we thank the Reviewer for her/his careful reading of the manuscript and for noting that the data and conclusions are clearly presented and scientifically sound. We respectfully disagree, however, with the assessment that the present work lacks novelty. To clarify the reason of this disagreement, we restate what we consider the core advance of the present study. Our main contribution here is not a *qualitative* study of the Johari–Goldstein–like process, nor the observation of its sub-diffusive nature, which have indeed been discussed previously by us and by others. Rather, the novelty of the present work lies in an experimental characterization of the crossover from quasi-localized to diffusive dynamics around Tc which has allowed us to establish a *quantitative* connection between the secondary (Johari–Goldstein) relaxation and the power-law increase of the mean-squared displacement prior to the onset of diffusion, thereby enabling a direct visualization of how diffusion emerges from the cage-rattling regime in a molecular liquid.
We agree with the Reviewer that the connection between the β_{JG}-relaxation and the cage rattling dynamics has been searched for a long time, in particular in the years during which the Mode Coupling Theory was proposed and compared to experimental results. However, this search - mainly based on a comparison of timescales - was largely unsuccessful, to the point that in became customary to talk in terms of *slow* and *fast* β processes, with the former ones associated to Johari-Goldstein processes and the latter ones to cage rattling dynamics. This distinction is still used in the literature. This should hopefully clarify, we think, the novelty of our results: we introduce a novel inversion scheme that allows associating *directly* and *quantitatively* the Johari-Goldstein relaxation to the cage rattling dynamics, surpassing the previous mentioned difficulties. To the best of our knowledge, this is the first time that such a point is made.
In other terms, while the present study surely and admittedly builds on earlier work, it goes substantially beyond previous publications, including our own ones, and leads to new, experimentally supported conclusions that were not accessible earlier. More precisely, in Refs. [19,30] (which correspond to the same publication; we apologize for this duplication and have corrected it in the revised manuscript), we demonstrated

qualitatively that in the undercooled liquid phase the Johari–Goldstein relaxation corresponds to a spatially restricted rearrangement of molecules within the cage of their nearest neighbors. In Ref. [35], we showed that this process is associated with fast, percolating spatio-temporal fluctuations. Neither work, however, provided a real-space, quantitative connection between the Johari–Goldstein relaxation and the onset of diffusion, nor its relationship with the α-relaxation, which are the central results of the present study.

1) However, the clarity could be improved by adding discussions regarding the effect of 5M2H being a mono-alcohol, where often another slower peak is seen in BDS data, possibly adding uncertainty regarding the interpretation of the main relaxation peak.

   Reply: we thank the reviewer for this comment. In addition to the α- and β_{JG}-relaxations, the dielectric spectra of monohydroxyl alcohols exhibit in fact the so-called Debye peak. This feature is intrinsic to the dielectric response and is generally absent in other response functions, such as density fluctuations (see the discussion in Ref. [19]) or in the orientational spectra measured by photon-correlation spectroscopy (PCS) [Gabriel et al., Phys. Rev. Lett. 121, 035501 (2018), new Ref. [57]]. Importantly, the activation barrier and characteristic timescales of the α-relaxation obtained from dielectric spectroscopy (where it is partially obscured by the Debye peak) and from PCS (where the Debye contribution is absent) are found to coincide (Ref. [57]). We can therefore conclude that the structural relaxation of 5M2H is well established and reliably determined. A comment along these lines has now been added to the manuscript in the discussion of the sample in the Methods section.

2) Overall, this work shows improved results concerning an interesting topic that deserves publication, but lacking the novelty required for Nature Physics.

   Reply: We thank the Reviewer for her/his careful review. As discussed, we respectfully maintain our point of view that the present study provides new and significant experimental insights that go clearly beyond previous work and therefore meets the novelty criteria of Nature Physics. We hope that the Reviewer, also in view of the discussion above, might reconsider her/his evaluation.

Reviewer 1

This manuscript presents a very interesting study on the relation between the alpha relaxation and the Johari Goldstein relaxation that comes from a well known group. The authors frame the discussion in a general context and then perform the measurements on a specific molecule, a typical glass former, the 5-methyl-2-hexanol, in its liquid supercooled state down to low supercooled temperatures. They use a wavevector-resolved x-ray technique to access microscopic, real-space information. The results are very interesting and certainly worth a Nature Physics but the interpretation would benefit from more discussion.

Reply: We thank the Reviewer for deeming our results interesting and worth publication in Nature Physics. In the following we address her/his concerns and comments point-by-point.

1) The alpha relaxation is a translational relaxation time. Does their spectroscopy measure only translations? Because they compare their results with dielectric relaxation that is a different cross section and measures rotations. I ask the authors to explain to the readers what quantities different cross sections measure, when comparing them.

   Reply: We thank the Reviewer for rising this point. Dielectric spectroscopy mainly probes molecular rotations, via electric dipole fluctuations; X-ray quasi-elastic scattering probes density fluctuations, as discussed in the Methods section, and can in principle be sensitive to both molecular translations and rotations. While we are not in position to disentangle these two contributions in our data, we observe that, depending on the probed wavenumber, density fluctuations couple to either the structural relaxation (at low q) or to the Johari-Goldstein relaxation (at high q). These processes are also observed in dielectric spectroscopy studies, from which we can conclude that electric dipole fluctuations and density fluctuations are coupled at around Tc in the sample studied here. A comment along these lines has now been added to the introduction of our manuscript.

2) Can the Johari Goldstein relaxation be associated to rotations, or do the authors think that is a translational process, or do they have no clue?

   Reply: The Johari Goldstein relaxation is surely associated to rotations, given its visibility in dielectric spectroscopy spectra. From our data and literature data we can conclude that the Johari-Goldstein relaxation is also coupled to both electric dipole and density fluctuations at high q. Most likely, at the relatively low temperatures explored here, rotations and translations are coupled, though a detailed MD simulation would be required to specifically address this point.

3) In water, a supercooled molecular liquid that follows Mode Coupling Theory, rotations and translations are coupled while molecules rattle in the cage and when the cage relaxes they decouple. See for example Chen et al, PRE 56, 4231 (1997) where this phenomenon is clearly shown. Do their conclusions on 5-methyl-2-hexanol support this picture?

   Reply: We thank the Reviewer for the interesting reference. As mentioned above, our measurements directly indicate that electric dipole fluctuations and density fluctuations are coupled both during molecular rattling in the cage (Johari-Goldstein relaxation) and during structural relaxation. In particular, we observe that density fluctuations couple to the Johari-Goldstein at high q where, according to the cited paper, roto-translational coupling is strong. Actually, the temperature range explored here (across Tc) is lower than that explored in the mentioned simulation work, so roto-translational coupling might be even stronger than in that case. Therefore,

it is possible that the Johari-Goldstein relaxation is mainly associated to rotations and that it is the roto-translational coupling, which is small at low-q, the mechanism that makes the Johari-Goldstein relaxation visible in the density fluctuations at high q, consistently with the mentioned simulation work. We added a comment along those lines in the results/discussion section of our manuscript.

Reviewer 2

The authors use nuclear gamma-resonance time-domain spectroscopy to gain insight into comparing the alpha and JG-beta relaxations in a hydrogen-bonded liquid, 5M2H. The data clearly reveal a change in temperature dependence at Tc, but more importantly a change in the q-dependence from diffusive to sub-diffusive motion, indicating that the beta-relaxation is the cage-rattling process, beyond which diffusion sets in. The data support the conclusions, but similar works by the same authors have arrived at the same overall conclusions [their ref. 19, 30, 35]. The present study is more detailed than previous ones, but lacks novelty regarding the conclusions. Also, previous work from other authors have indicated this nature of the beta-relaxation observed here. The paper and data presentation are very clear, with appropriate references.

Reply: we thank the Reviewer for her/his careful reading of the manuscript and for noting that the data and conclusions are clearly presented and scientifically sound. We respectfully disagree, however, with the assessment that the present work lacks novelty. To clarify the reason of this disagreement, we restate what we consider the core advance of the present study. Our main contribution here is not a *qualitative* study of the Johari–Goldstein–like process, nor the observation of its sub-diffusive nature, which have indeed been discussed previously by us and by others. Rather, the novelty of the present work lies in an experimental characterization of the crossover from quasi-localized to diffusive dynamics around Tc which has allowed us to establish a *quantitative* connection between the secondary (Johari–Goldstein) relaxation and the power-law increase of the mean-squared displacement prior to the onset of diffusion, thereby enabling a direct visualization of how diffusion emerges from the cage-rattling regime in a molecular liquid.
We agree with the Reviewer that the connection between the β_{JG}-relaxation and the cage rattling dynamics has been searched for a long time, in particular in the years during which the Mode Coupling Theory was proposed and compared to experimental results. However, this search - mainly based on a comparison of timescales - was largely unsuccessful, to the point that in became customary to talk in terms of *slow* and *fast* β processes, with the former ones associated to Johari-Goldstein processes and the latter ones to cage rattling dynamics. This distinction is still used in the literature. This should hopefully clarify, we think, the novelty of our results: we introduce a novel inversion scheme that allows associating *directly* and *quantitatively* the Johari-Goldstein relaxation to the cage rattling dynamics, surpassing the previous mentioned difficulties. To the best of our knowledge, this is the first time that such a point is made.
In other terms, while the present study surely and admittedly builds on earlier work, it goes substantially beyond previous publications, including our own ones, and leads to new, experimentally supported conclusions that were not accessible earlier. More precisely, in Refs. [19,30] (which correspond to the same publication; we apologize for this duplication and have corrected it in the revised manuscript), we demonstrated

qualitatively that in the undercooled liquid phase the Johari–Goldstein relaxation corresponds to a spatially restricted rearrangement of molecules within the cage of their nearest neighbors. In Ref. [35], we showed that this process is associated with fast, percolating spatio-temporal fluctuations. Neither work, however, provided a real-space, quantitative connection between the Johari–Goldstein relaxation and the onset of diffusion, nor its relationship with the α-relaxation, which are the central results of the present study.

1) However, the clarity could be improved by adding discussions regarding the effect of 5M2H being a mono-alcohol, where often another slower peak is seen in BDS data, possibly adding uncertainty regarding the interpretation of the main relaxation peak.

   Reply: we thank the reviewer for this comment. In addition to the α- and β_{JG}-relaxations, the dielectric spectra of monohydroxyl alcohols exhibit in fact the so-called Debye peak. This feature is intrinsic to the dielectric response and is generally absent in other response functions, such as density fluctuations (see the discussion in Ref. [19]) or in the orientational spectra measured by photon-correlation spectroscopy (PCS) [Gabriel et al., Phys. Rev. Lett. 121, 035501 (2018), new Ref. [57]]. Importantly, the activation barrier and characteristic timescales of the α-relaxation obtained from dielectric spectroscopy (where it is partially obscured by the Debye peak) and from PCS (where the Debye contribution is absent) are found to coincide (Ref. [57]). We can therefore conclude that the structural relaxation of 5M2H is well established and reliably determined. A comment along these lines has now been added to the manuscript in the discussion of the sample in the Methods section.

2) Overall, this work shows improved results concerning an interesting topic that deserves publication, but lacking the novelty required for Nature Physics.

   Reply: We thank the Reviewer for her/his careful review. As discussed, we respectfully maintain our point of view that the present study provides new and significant experimental insights that go clearly beyond previous work and therefore meets the novelty criteria of Nature Physics. We hope that the Reviewer, also in view of the discussion above, might reconsider her/his evaluation.

Reviewer 1

The authors have replied to all the points that I raised satisfactorily and the current version of the paper is now further improved. For me it can be accepted in the present form.

Reply: We thank the reviewer for their positive assessment of our revised manuscript. We are pleased that our responses have satisfactorily addressed all concerns and that the improvements are appreciated.

Reviewer 2

With the additional explanations in the response letter and the changes in the text, I feel the authors have acted convincingly on the criticism of both reviewers. In its revised form, the paper is now recommended for publication in Nature Physics.

Reply: We sincerely appreciate Reviewer 2's recognition of our efforts to improve the manuscript in response to their advice, and we thank them for their support of its publication.
